# Mixed Reality Biopsy Navigation System Utilizing Markerless Needle Tracking and Imaging Data Superimposition

**DOI:** 10.3390/cancers16101894

**Published:** 2024-05-16

**Authors:** Michał Trojak, Maciej Stanuch, Marcin Kurzyna, Szymon Darocha, Andrzej Skalski

**Affiliations:** 1 Department of Measurement and Electronics, AGH University of Krakow, 30-059 Krakow, Poland; trojak@agh.edu.pl; 2 MedApp S.A., 30-150 Krakow, Poland; maciej.stanuch@medapp.pl; 3 Department of Pulmonary Circulation, Thromboembolic Diseases and Cardiology, Centre of Postgraduate Medical Education, European Health Centre, 05-400 Otwock, Poland; marcin.kurzyna@ecz-otwock.pl (M.K.); szymon.darocha@ecz-otwock.pl (S.D.)

**Keywords:** biopsy, image-guided percutaneous needle biopsy, augmented reality, mixed reality, computer vision, surgical navigation

## Abstract

**Simple Summary:**

Ensuring precise needle placement during biopsy procedures is essential for both successful outcomes and minimizing patient risk. Our study presents a new mixed reality system that helps doctors accurately navigate needles to their intended targets without needing physical markers. By using advanced imaging and computer vision techniques, the system overlays anatomical data directly onto the patient, guiding the needle along a pre-planned path. We tested this system in various ways, including its accuracy and efficiency in needle placement. Our findings showcased a significant improvement, with a reduction in number of punctures needed to reach the target location. The test was successfully completed on the first attempt in 70% of cases, as opposed to only 20% without the system. Additionally, there was a 53% reduction in procedure time, validating the effectiveness of the system.

**Abstract:**

Exact biopsy planning and careful execution of needle injection is crucial to ensure successful procedure completion as initially intended while minimizing the risk of complications. This study introduces a solution aimed at helping the operator navigate to precisely position the needle in a previously planned trajectory utilizing a mixed reality headset. A markerless needle tracking method was developed by integrating deep learning and deterministic computer vision techniques. The system is based on superimposing imaging data onto the patient’s body in order to directly perceive the anatomy and determine a path from the selected injection site to the target location. Four types of tests were conducted to assess the system’s performance: measuring the accuracy of needle pose estimation, determining the distance between injection sites and designated targets, evaluating the efficiency of material collection, and comparing procedure time and number of punctures required with and without the system. These tests, involving both phantoms and physician participation in the latter two, demonstrated the accuracy and usability of the proposed solution. The results showcased a significant improvement, with a reduction in number of punctures needed to reach the target location. The test was successfully completed on the first attempt in 70% of cases, as opposed to only 20% without the system. Additionally, there was a 53% reduction in procedure time, validating the effectiveness of the system.

## 1. Introduction

Image-guided percutaneous needle biopsy (PNB) is a first step in establishing a diagnosis, and plays a pivotal role in guiding subsequent treatment decisions. In contrast to open or excisional biopsy procedures, image-guided PNB is a minimally invasive option that can often be recommended as an outpatient service for the majority of cases [1]. It is also valuable for detecting residual or recurrent post-treatment diseases [2].

The choice of imaging technique relies on factors such as the type and location of the lesion, patient compliance, the availability of the technique, and the preferences of the operators [1]. One of the most popular supporting technique is ultrasound (US) imaging. Its advantages include fast acquisition time, real-time needle evaluation, lack of radiation exposure, avoidance of vessels through color Doppler imaging, availability, shorter procedure time, and decreased cost [3,4]. On the other hand, the feasibility of this method depends highly on operator experience and the presence of an adequate acoustic window, which is essential for visualizing the lesion [5]. The outcome of the procedure is also influenced by factors such as the size of the lesion (with lesions of size 20 mm or greater considered adequate), location of the lesion and its distance to the skin, and type of lesion, with certain types having lower diagnostic success rates compared to surgical biopsy [6]. An ongoing issue with this method is poor needle visibility [7].

Another widely used technique is Computer Tomography (CT)-guided biopsy. It offers exceptional contrast and high spatial resolution, facilitating optimal needle visualization and precise positioning within the target lesion [8]. It is very versatile, and can be applicable across various organs, ranging from superficial soft tissue lesions to deep-seated lesions within the thoracic or abdominal cavities [9]. However, this method is associated with significant risk of complications; the most common are pneumothorax and pulmonary hemorrhaging, along with exposure to radiation [4,10]. There are plenty of risk factors for the development of complications as well, including increased number of punctures, wider insertion angle of the needle, small lesion size, greater lesion depth, and long biopsy path [11]. What is more, real-time imaging is possible only with fluoroscopy [12].

Last but not least, Magnetic Resonance (MR) is another popular biopsy guidance imaging system. This method provides excellent soft tissue contrast, does not use ionizing radiation, enables continuous visualization of vessels throughout a procedure without the need for intravenous contrast, and has a distinctive ability to elicit different tissue characteristics during a procedure by using different pulse sequences [13]. This technique is employed if lesions are not identifiable by mammography or ultrasonography [14], and is challenging when there are difficulties around lesion access, validating sufficient lesion sampling, and establishing accurate radiologic–pathologic correlation for enhancing lesions [15]. Moreover, due to the presence of a strong magnetic field, the needles must meet electromagnetic compatibility requirements [16]. A summarization of the advantages and disadvantages of each method is presented in Table 1.

In order to overcome mentioned issues, we propose an extension to guidance systems that exploits mixed reality without external trackers. It is designed to guide the operator through the biopsy procedure with potentially enhanced certainty, precision, and efficiency, resulting in shorter procedural times. The system consists of superimposing patients’ 3D-reconstructed diagnostic CT or MR scan onto the body and performing needle tracking through a combination of hybrid deep learning and deterministic methods. The results are visualized in holographic space using a uniform coordinate system that consists of a Head-Mounted Display (HMD), patient, and needle. This study aims to validate the proposed approach by assessing the needle injection precision, time required for procedure execution, and minimum adequate size of nodules.

Mixed reality is a rapidly growing area of research in different medical specializations. Glasses can be used to display information that might be beneficiary to the operator, such as a stream of 2D video in holographic space, 3D volumetric visualization of the patient’s body, or direct overlay of the patient, which is not possible with standard displays. Systems based on mixed reality have already been used in cardiac interventions and cardiac surgery [17,18,19,20], orthopaedics [21,22,23], vascular surgery [24,25], and surgical oncology [26].

Artificial intelligence can be an important supporting tool for mixed reality systems in the medical technology field. It is currently being utilized in various specialities, including oncology, radiology, radiotherapy, and surgery [27]. Deep learning models can be used to, e.g., detect cancer at an early stage [28], improve image quality, decide on important imaging examinations [29], predict spatial dose distribution [30], and automatically identify surgical phases or instruments [31]. An example of AI application is biopsy needle segmentation, which is a part of the proposed solution.

Related work is described in the following section.

### Related Work

A comparative study on Augmented Reality (AR) navigation for cranial biopsy was conducted by Skyrman et al. [32], who examined a system utilizing a ceiling-mounted robotic C-arm with intraoperative Cone–Beam CT (CBCT) capability. It integrates an optical tracking system with four cameras into the C-arm’s flat panel for co-registration, patient and instrument tracking, and image augmentation. The study involved using a CT scan obtained from the DICOM online library as a basis for creating a printed 3D model that served as a phantom. It was additionally equipped with 30 steel balls (2 mm ± 5 µm diameter) to indicate targets for injections. Subsequently, the phantom was scanned using CBCT for registration and planning of biopsy paths. The operators were guided during the procedure by a three-dimensional needle tracking navigation system. For testing purposes, thirty individual biopsy insertions were performed. After needle positioning, a new CBCT scan was performed for verification of the needle position. The accuracy was established by measuring the distance between the tip of the inserted needle and the center of the target while subtracting the ball radius of 1 mm. The measurements also concerned the navigation time following the planned path and the correlation between path length and accuracy. The median path length was 39 mm (range 16–105 mm); our study examines more distant target locations. Moreover, the examination did not involve measuring the repeatability of the procedure. No significant correlation was detected between path length and accuracy; however, accuracy testing involving more distant locations could provide different results due to the angular error. The execution time measurements were not compared to results without using the system to establish its usability and impact on potentially reducing the procedure time. Finally, the study did not establish potential reduction of punctures needed to hit the target.

Another study compared the technical feasibility of an AR navigation system with a standard CT-guided approach [33]. It utilized markers to superimpose patients’ reconstructed anatomical structures and needle tracking. Testing on human subjects involving eight patients (four males) aged 58 ± 24 years (mean ± standard deviation) was performed in a group. Biopsies were performed using the AR solution; for another eight patients (four males) aged 60 ± 15 years, biopsies were guided using CT. For each procedure, the collected data were as follows: elapsed time from local anesthesia to specimen withdrawal, number of CT passes, radiation dose, complications, specimen adequacy, and the height, weight, and body mass index of the AR group patients. No complications were observed in either group, there were no significant differences in terms of procedure duration, and every procedure was completed as initially planned, reaching the target lesion and obtaining a bioptic sample. However, the number of CT passes and radiation dose were significantly lower when using the AR solution. The study did not present quantitative data about path length, which could be valuable due to the possibility of angular error. The number of needed punctures was not indicated, which could provide information about the likelihood of reducing risk of post-procedural complications. Finally, the study did not precisely describe the a placement of the target spots or whether the individual procedures had greater risk of complications.

## 2. Materials and Methods

This section describes the components of the proposed mixed reality system. It is divided into two parts: Section 2.1 describes the technical aspects of the developed solution, while Section 2.2 presents the clinical workflow from the operator’s perspective.

### 2.1. Proposed Solution

**Figure 1:**
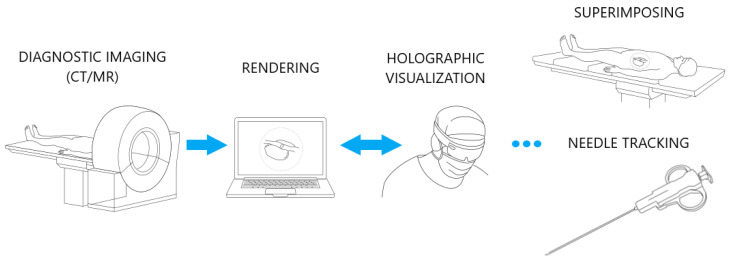
Biopsy navigation system scheme.

The biopsy navigation system scheme is presented on Figure 1. In a first step, a patient diagnostic scan is obtained using CT or MR imaging and saved in DICOM file format. The data are 3D-reconstructed, rendered on a work station using CarnaLife Holo technology [26], and visualized on a HoloLens 2 headset. The HMD is equipped with a set of sensors and cameras which are accessible by Research Mode [34]. This enables the possibility of capturing physical objects and mapping them into holographic space. Devices share data with each other within a local network. The visualized data are superimposed onto the patient’s body while preserving the real dimensions of the scan. The operator selects a desired optimal trajectory along which to perform the injection. To ensure injection precision and certainty, we have devised a needle tracking module. This module guides the operator to align the needle with the designated trajectory line as closely as possible.

**Figure 2:**
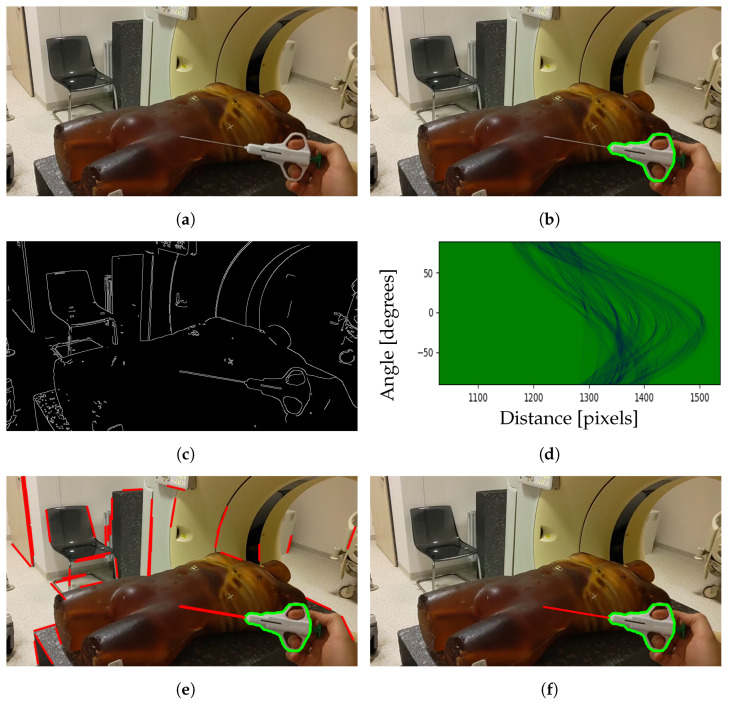
Steps for segmenting biopsy needle from image: (**a**) image captured by the PV camera; (**b**) segmented tool region; (**c**) detected edges; (**d**) Hough space; (**e**) detected lines; (**f**) chosen line belonging to the needle.

The needle tracking method is presented in Figure 2. It involves the acquisition of images through a Photo–Video (PV) camera and identification of the tool region using the YOLOv8 model [35] for instance segmentation as a first step, shown in Figure 2a and Figure 2b, respectively. As the needle itself is not well recognized by the model, it is trained only to segment the grabber part of a tool. Subsequently, the needle line is established through the application of edge detection and the Hough transform. The thresholds for the edge detection are adjusted using a Grey Wolf Optimizer metaheuristic algorithm [36]. A binary image displaying the detected edges is shown in Figure 2c.

The Hough transform is used for finding line segments in a set of points. It transforms every point coordinate in image space (x,y) into a set of lines (r,θ) using parametric notation:

(1) $$\begin{array}{c} x\cos\theta +y\sin\theta =r \end{array}$$

where *r* is the length of a normal from the origin to the line and θ is te angle of the line with respect to the horizontal axis. Mapping all possible (r,θ) values for points given by (x,y) in Cartesian image space results in curves (i.e., sinusoids) in Hough parameter space, as presented in Figure 2d. A set of two or more points that form a straight line will produce sinusoids crossing at a given (r,θ) for that line. The line is selected if the number of intersections of sinusoids is greater than the given threshold [37]. This results in a series of lines, as presented in Figure 2e. Among them, one line is chosen that best matches the segmented tool region and the motion of the previous positions. The final result of needle segmentation is presented in Figure 2f.

**Figure 3:**
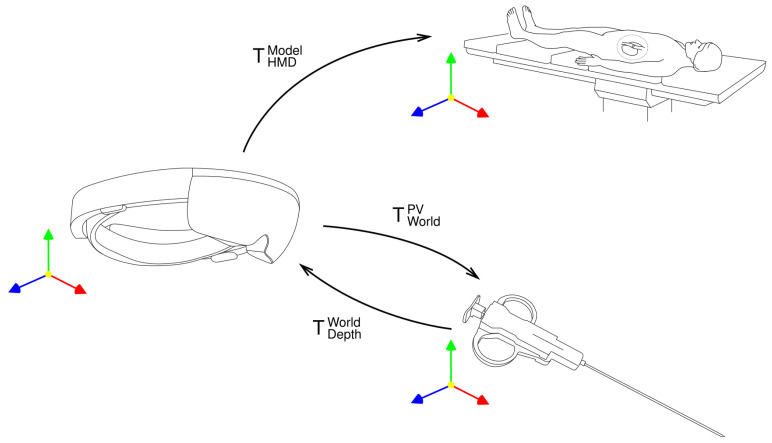
Transformations between coordinate systems.

To attain the 3D pose of the tool in holographic space, the segmented region of interest is aligned with a part of the point cloud obtained by the depth camera. The depth camera operates in two modes: Articulated Hand Tracking (AHAT), offering high-frequency (45 FPS) near-depth sensing up to 1 m, and Long-Throw, providing low-frequency (1–5 m) far-depth sensing. Due to the superior performance of the camera in AHAT mode and the fact that the needle is in range of 30–60 cm from the operator, this mode was chosen. To achieve coordination between the PV and depth cameras, their respective coordinate systems were interconnected through a transformation process in order to align them with the world coordinate system of the headset. These transformations between coordinate systems are presented on Figure 3.

The point cloud acquired by the depth camera is converted to HMD world coordinates by multiplying the points by the inversed extrinsics matrix [38]:

(2) $$\begin{array}{c} T_{Depth}^{World}={\left[\begin{array}{ccc} R & | & t \end{array}\right]}_{Depth}^{-1} \end{array}$$

where *R* denotes 3D rotation and *t* denotes 3D translation. To obtain the related pixel coordinates in an image from the PV camera for a given point in the world coordinates, it is multiplied by the camera matrix, resulting in its position in the image coordinate system [38]:

(3) $$\begin{array}{c} T_{World}^{PV}=K_{PV}{\left[\begin{array}{ccc} R & | & t \end{array}\right]}_{PV} \end{array}$$

(4) $$\begin{array}{c} K=\left[\begin{array}{cccc} f_{x} & 0 & c_{x} & 0\\ 0 & f_{y} & c_{y} & 0\\ 0 & 0 & 1 & 0 \end{array}\right] \end{array}$$

where *K* denotes the camera’s intrinsic matrix and $\left[\begin{array}{ccc} R & | & t \end{array}\right]$ denotes the camera’s extrinsic matrix. The intrinsic matrix parameters $f_{x}$ and $f_{y}$ are the focal length of the camera in the *x* and *y* dimensions, respectively, while $c_{x}$ and $c_{y}$ denote the principal point of the camera expressed in pixels. The position of the projected point in pixel coordinates can then be calculated as follows:

(5) $$\begin{array}{c} x_{i}=\left\lfloor \frac{x_{i}}{z_{i}}\right\rfloor ,y_{i}=\left\lfloor \frac{y_{i}}{z_{i}}\right\rfloor \end{array}$$

where $x_{i}$, $y_{i}$, and $z_{i}$ are point coordinates in the image coordinate system.

The needle itself is invisible to the depth camera; consequently, it is estimated based on the grabber part of the tool. Due to high distortion of the point cloud, the needle’s pose is determined by selecting a set of points that most accurately align with the needle line, filtering out outliers, and conducting a linear regression. To enhance the stability of needle estimation, the Kalman filtering and exponential moving average techniques are employed.

To obtain needle’s pose relative to the data, the coordinate systems of applications running on the HMD and workstation need to be synchronized. Thus, the line segment forming the needle is successively transformed into model coordinates:

(6) $$T_{HMD}^{Model}={\left[\begin{array}{cccc} sR_{11} & sR_{12} & sR_{13} & t_{x}\\ sR_{21} & sR_{22} & sR_{23} & t_{y}\\ sR_{31} & sR_{32} & sR_{33} & t_{z}\\ 0 & 0 & 0 & 1 \end{array}\right]}_{M}{\left[\begin{array}{cccc} R_{11} & R_{12} & R_{13} & t_{x}\\ R_{21} & R_{22} & R_{23} & t_{y}\\ R_{31} & R_{32} & R_{33} & t_{z}\\ 0 & 0 & 0 & 1 \end{array}\right]}_{C}$$

where matrix the with subscript *C* denotes transformation between the coordinate systems of the devices, the matrix with subscript *M* denotes transformation to the model coordinates, *R* denotes 3D rotation, *t* denotes 3D translation, and *s* is a scale factor.

### 2.2. Clinical Workflow

**Figure 4:**
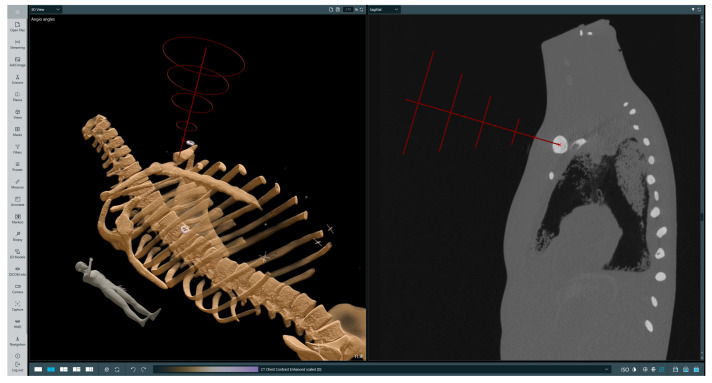
Selected trajectory visualization. The **left-hand** image presents a volumetric rendering of the phantom’s CT scan with the chosen trajectory, while the **right-hand** image presents a 2D slice in the sagittal plane with the indicated injection site and target location. On the trajectory (red line) the first red cross symbol indicated injection point, the second landing point.

The initial step of biopsy procedure planning is selecting a trajectory along which the injection will be performed. The operator first selects a point at the target location, then a point on a skin at the injection site; afterwards, a straight line is drawn from the target spot through the injection site and above the patient’s body. There are several rings around the designated line, which are intended to guide the operator by appropriately highlighting the needle’s position in relation to the trajectory. After the trajectory is set, it can be revised by the operator in both the 3D and 2D views. This is a crucial point, at which the operator verifies the trajectory and ensures that the incision will not endanger any important organs. A sample selected trajectory is presented on Figure 4.

**Figure 5:**
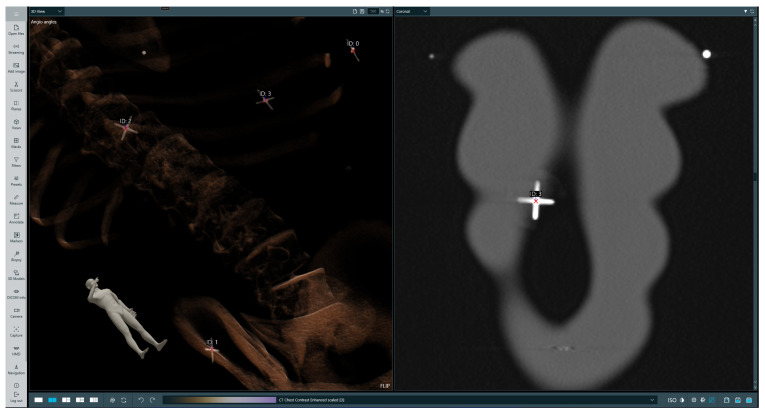
Selected markers in the data. The **left-hand** image presents a volumetric rendering of the phantom’s CT scan, while the **right-hand** image presents the 2D slice in the coronal plane with radiological marker.

**Figure 6:**
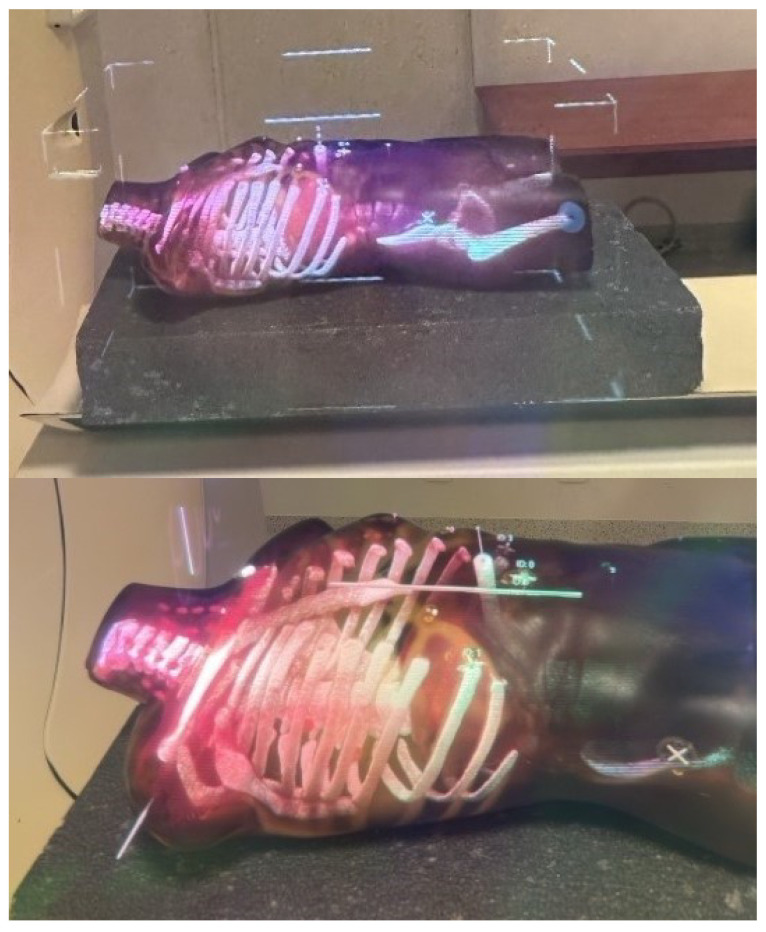
Data superimposition captured from HMD lens.

The subsequent phase involves superimposing data within the holographic space directly onto the patient’s body. To perform this process, the patient undergoes scanning with radiological markers strategically placed in specific locations of the body surface that serve as reference points and are clearly visible in the image data across multiple slices. The operator begins by selecting each marker on 2D slices, assigning them unique identifiers, and then selecting corresponding physical markers in the holographic space. Sample selected markers are presented in Figure 5. Afterwards, the hologram representing the patient’s anatomy is transformed to minimize the distance between corresponding physical and virtual markers while preserving the real scale of the patient scan. The result of superimposing the data captured by the HMD lens is presented in Figure 6.

**Figure 7:**
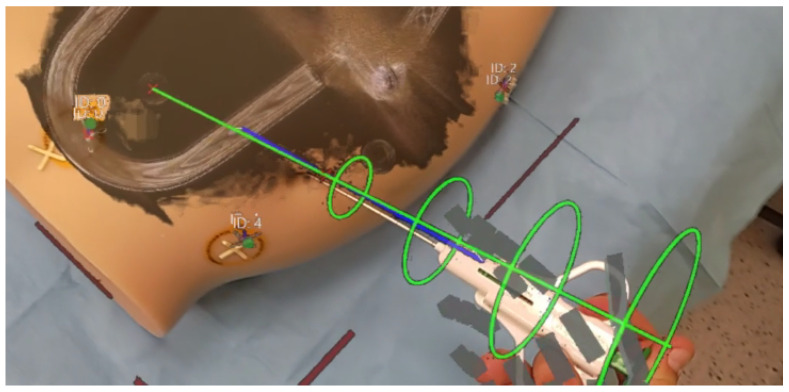
Needle tracking interface on HMD.

The final step encompasses needle tracking to achieve precise alignment with the chosen trajectory. The rings and trajectory line dynamically change colors based on the needle’s alignment accuracy. Additionally, the operator can perceive guiding lines extending from both needle ends to the trajectory line, providing a visual indication of the needle’s proximity to the intended path. The HMD needle tracking interface is presented in Figure 7.

When selecting a mixed reality headset for surgical environments, careful consideration of the operating room’s unique demands is essential. The primary options are see-through and video pass-through headsets. See-through headsets allow for direct viewing of the surgical field through transparent glass, overlaying virtual elements onto this view. In contrast, video pass-through headsets relay external camera feeds onto internal screens, potentially introducing latency and visual disturbances.

See-through headsets offer the precise real-time visualization crucial for surgical accuracy while maintaining uninterrupted visual contact between surgeon and patient, facilitating seamless communication within the surgical team. Additionally, see-through headsets streamline the integration of augmented reality overlays, enhancing surgical precision. Therefore, in the context of surgical procedures, see-through headsets stand out as the preferred choice for optimal performance and compatibility. In this study, we have chosen the HoloLens 2 headset, as it is suitable for use by a clinicans in the operating room.

## 3. Results

This section describes the results of the needle pose estimation accuracy test, described in Section 3.1, and three types of tests performed on phantoms using the developed solution: the final location error of the injected needle, described in Section 3.2; the material collection efficiency for an imitation lesion, described in Section 3.3; and the time needed to conduct the injection, described in Section 3.4.

The obtained needle tracking refresh rate was 20 Hz. The algorithm was implemented in C++, and the system ran on a workstation with an Nvidia RTX3080 graphics card and AMD Ryzen 9 5950X processor. The overall performance proved feasible for real-time operations.

### 3.1. Needle Pose Estimation Accuracy

This test involved detecting two needles sized 120 mm and 160 mm standing vertically in front of the camera and rotated 15° around needle axis at four different distances from the headset. Subsequently, the displacement of both the base point at the grabber and the tip of the needle from their algorithmically determined positions was measured using a caliper. The results are presented in Table 2 and Table 3. It is noticeable that the offset from the physical needle tip increases significantly in the case with the needle oriented frontally, as the needle is too close or too far from the camera. When rotating the needle horizontally, the accuracy increases at shorter ranges but decreases at greater distances. A higher needle tip error in comparison to the base error results when determining its position based on the grabber part of the tool, which is a needle’s length away.

### 3.2. Final Location Error

**Figure 8:**
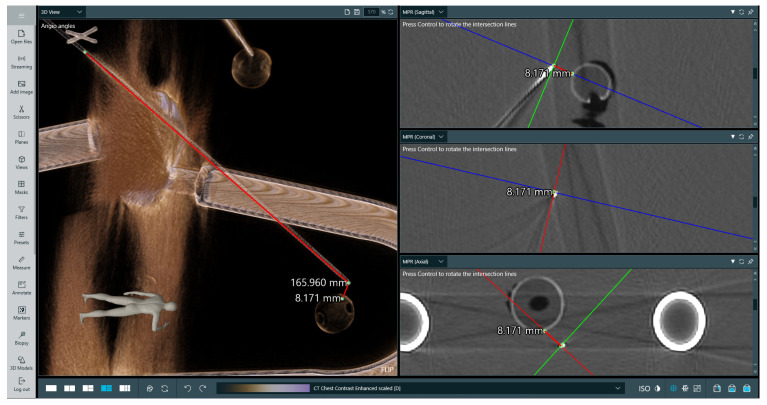
Measurement of puncture depth and distance to imitation lesion for examination no. 3. The target was at a depth of 16 cm and the measurement was validated using CT. The **left-hand** image presents a volumetric rendering of the phantom’s CT scan and the performed measurements, while the **right-hand** image presents three 2D slices in perpendicular planes with marked measurements between the needle tip and the lesion. On the **right-hand** side image, lines indicate the intersection between anatomical planes.

This test involved using two types of phantoms: a professionally made radiological one with imitation lesions located at different places, and a deformable one made from cyberskin with embedded aluminium spinal cord and inserted wooden balls to simulate lesions. The phantoms underwent CT scanning to perform superimposition with the obtained imaging data. Subsequently, a biopsy procedure was performed to hit the surface of an imitation lesion using the developed system. The phantoms then underwent another round of CT scanning to measure the distances between the injected needle positions and designated target locations in the data. The results of the measurements are presented in Table 4. As can be observed, while the location error increases when the lesion is located deeper, it remains appropriate. It is important to note that some needle deflection occurred, as it was tailored for softer materials. The results of performed measurement no. 3 are presented in Figure 8.

### 3.3. Material Collection Efficiency

This test involved using the deformable phantom made of cyberskin with inserted imitation lesions. It was examined by puncturing two imitation lesions of varying sizes 24 times each to assess the accuracy of the procedure. The study was conducted by a physician with over three years of experience in procedures requiring needle puncture of the body. Access to the lesions took place from different sides. Each time, the physician pulled the needle out completely and performed the injection again. The feedback on whether the target was hit was a feeling of resistance. The results of the test are presented in Table 5. Examination no. 2 was performed on a radiological phantom, while the others were performed on a deformable one. The accuracy for both lesions was very high, reaching 100% for the larger one.

### 3.4. Injection Time

This test was performed by ten physicians, of whom six had at least 3 years of experience in biopsy or related procedures and four were less experienced. The test compared procedures performed on a deformable phantom while using the proposed system and while using only a displayed CT image in terms of execution time and number of punctures needed. The test results are presented in Table 6. Use of the system led to a 53% reduction in time needed for preparation and to perform the biopsy procedure on the phantom. Moreover, the operator had to perform fewer injections on average when using the proposed system. The test procedure was completed on the first attempt in 70% of cases, in comparison to 20% without the system. The only case in which the procedure execution time increased was during examination no. 8, which was due to insufficient training before attempting the test.

## 4. Discussion

This section includes an analysis of the results in Section 4.1, a discussion of the advantages of the system in Section 4.2, and a discussion of its limitations in Section 4.3.

### 4.1. Results Analysis

The test results clearly indicate that the implemented solution can reduce the number of required punctures, as in most cases the physician completed the procedure on the fist attempt when using the system. This leads to a lower likelihood of post-procedural complications, reducing blood loss and the risk of damaging organs, and contributes to faster patient recovery. Importantly, proper training and familiarization with the system is crucial to ensure better results. Participants that took the time to familiarize themselves with how to operate the system obtained better results.

This study revealed that while utilizing the developed system there was no significant difference in biopsy procedure execution times between less experienced physicians, who demonstrated a 60% improvement, and more experienced ones, who achieved a 48% enhancement. These results indicate that use of our solution is beneficial for operators at any level of expertise. Notably, competitive image guidance techniques such as US are highly dependent on operator experience, whereas in our case it is not especially relevant.

The accuracy of the procedure is potentially higher when dealing with shallower lesion depths due to the greater risk of angular error. It is imperative for the operator to precisely align the needle with the planned trajectory, as even minor deviations can result in a relatively substantial error when the lesion in located at a greater depth. However, the gathered results indicate exceptional accuracy, with a final location error below 1 cm for lesions at depths above 10 cm, confirming the usefulness of a solution. Furthermore, the repeatability of these results is proven the conducted material collection efficiency test.

The needle pose estimation accuracy test showcases that while markerless needle tracking is feasible, it requires an individual approach for particular tools in order to overcome issues with distorted point clouds and depth misalignments [39]. When looking straight at the needle, the average displacement error is at most 2.60 mm at a range of 50 cm from the headset. The best accuracy regardless of rotation is achieved when the operator is looking at the needle from about 40 cm, which is the most common case, probably due to the AHAT mode being designed for hand tracking [34]. This study reveals that the needle tracking method is useful for physicians when performing biopsy procedures.

### 4.2. Advantages of the Proposed Approach

The developed solution works on a local network, allowing it to function without the need for internet access, which is often a challenge in operating rooms. Such an approach improves accessibility, enhances security, reduces the risk of sensitive data leaks, and prevents connection delays.

By utilizing a see-through mixed reality headset, the operator perceives the real world directly, instead of projecting an image from a camera as in popular AR headsets. This helps to ensure a sterile working environment. Unlike common methods such as using glued or attached markers to track the biopsy needle [40], our solution employs a fully vision-based approach using only the headset. Thanks to this, it maintains the tool’s center of a mass and does not require any external tracking systems or cameras, thereby eliminating the need for a calibration procedure and making the whole system more intuitive and easier to use. From a clinical perspective, systems relying on external trackers or cameras are less effective during short procedures, as they require each component to be established.

Notably, physicians participating in the study highlighted that system usage, including data superimposition and trajectory planning, can shorten the preparation time needed prior to performing the procedure. In particular, overlaying a 3D model onto the patient’s body make it possible to visualize surgical targets as well as any organs at risk. Another significant factor is receiving feedback if the needle is aligned with the intended trajectory. The operator is guided to adjust the needle’s position, which enhances confidence. For US guidance techniques, it is necessary to inject the needle first in order to see it in an image, while our system navigates to ensure proper needle placement and angle before the injection. Importantly, unlike CT or MRI, the proposed system does not utilize any radiation or expensive intraoperative equipment during procedure, making it a safer and cost-effective alternative for medical interventions.

### 4.3. Limitations

It is crucial to acknowledge the system’s limitation in terms of its ability to adapt to tissue deformations. The patient’s scan remains insensitive to factors such as pressure, breathing, injection, and bladder filling. To address this problem, integrating supplementary imaging techniques such as US may be a viable solution. Although the developed needle tracking method proves effective if the needle is partially visible, further efforts are required to accurately track its position within the patient’s body and estimate the injection depth.

This study’s main limitation is the use of phantoms. While on the one hand this allows the error to be estimated precisely, on the other it does not take into account the time between the CT scan and the biopsy, which for humans is the most important factor. If the time difference is greater, the deformation of the actual state compared to the scanned one can be significant. In conclusion, future work should be extended to cadavers and human subjects.

## 5. Conclusions

This study has showcased that utilizing a markerless needle tracking method working without external cameras is feasible, yet requires an individual approach for particular tools. One of the biggest challenges in obtaining needle 3D pose is high distortion of the point cloud obtained from the HMD depth camera. However, such an approach is still more useful and universal than standard marker-based solutions. In addition, superimposing a 3D-reconstructed CT/MR scan containing the highlighted planned trajectory onto the patient’s body provides relevant information for the operator.

This study has proven that using a mixed reality navigation system in biopsy procedures is beneficial for physicians at any level of expertise. The most valuable test results are those finding a significant decrease in the number of punctures needed and reduced procedure time, which together can lead to faster patient recovery and minimize the risk of complications. The most crucial step for operators is to precisely inject the needle as intended, as even minor mistakes can lead to a major offset. Our system addresses this issue by allowing the operator to meticulously plan the procedure and by guiding them to align the needle with the selected trajectory.

## Figures and Tables

**Table 1:** Advantages and disadvantages of image guidance techniques used for biopsy procedures.

Modality	Advantages	Disadvantages
US	Fast acquisition time Real-time needle evaluation Lack of radiation exposure Vessel avoidance (Doppler) Shorter procedure time Less expensive	Operator experience-dependent Poor needle visibility Suitable acoustic window needed Reliant on lesion type, size, and location
CT	Exceptional contrast High spatial resolution Applicable across various organs	Higher risk of complications Exposure to radiation Fluoroscopy for real-time imaging
MR	High soft tissue contrast No ionizing radiation Vessel visualization without contrast Able to elicit tissue characteristics	Challenging lesion access Difficult lesion sampling verification Tough radiology–pathology matching Compatible needles needed

**Table 2:** Results of the needle pose estimation test when the tool is placed in front of the camera. The error is provided by the mean value ± standard deviation of ten measurements.

Distance from HMD [cm]	Needle 120 mm		Needle 160 mm	
	Needle Base Error [mm]	Needle Tip Error [mm]	Needle Base Error [mm]	>Needle Tip Error [mm]
30	1.53 ± 0.70	3.25 ± 1.59	0.89 ± 0.49	3.25 ± 1.43
40	1.47 ± 0.50	1.61 ± 0.95	1.19 ± 0.46	2.52 ± 1.04
50	1.16 ± 1.00	2.24 ± 1.06	0.87 ± 0.23	2.60 ± 1.38
60	2.68 ± 1.18	2.36 ± 1.37	2.80 ± 0.43	3.20 ± 1.63

**Table 3:** Results of the needle pose estimation test when the tool is rotated 15° around the needle axis. The error is provided by the mean value ± standard deviation of ten measurements.

Distance from HMD [cm]	Needle 120 mm		Needle 160 mm	
	Needle Base Error [mm]	Needle Tip Error [mm]	Needle Base Error [mm]	Needle Tip Error [mm]
30	1.08 ± 0.25	1.25 ± 0.75	1.04 ± 0.37	1.76 ± 0.82
40	0.88 ± 0.23	1.77 ± 0.64	0.87 ± 0.24	2.56 ± 0.96
50	1.63 ± 0.52	3.36 ± 1.37	1.65 ± 0.48	3.55 ± 1.07
60	3.12 ± 1.04	2.05 ± 1.01	4.23 ± 0.75	3.21 ± 2.19

**Table 4:** Results of the final location error test.

Exam No.	Puncture Depth [mm]	Distance to Lesion [mm]
1	62.39	0
2	109.15	9.74
3	165.96	8.17

**Table 5:** Results of material collection efficiency test.

Lesion Diameter [cm]	Distance to Lesion Range [cm]	Correct Punctures No.	Accuracy
5	4–10	24	100%
2	3.6–8	23	95.83%

**Table 6:** Results of the injection time test.

Exam No.	Experience >3 Years	With System		Without System		Time Difference
		Punctures No.	Total Time	Punctures No.	Total Time	
1	NO	1	03:08	2	09:13	66%
2	NO	1	00:09	1	00:17	47%
3	NO	2	00:42	4	01:27	52%
4	YES	1	00:17	3	01:08	75%
5	YES	1	00:14	4	01:37	86%
6	YES	1	00:07	1	00:13	46%
7	YES	1	00:40	2	01:21	51%
8	YES	3	02:31	3	02:14	−13%
9	NO	1	00:06	2	00:23	74%
10	YES	2	00:57	3	01:42	44%
Average		1.4	00:53	2.5	01:57	53%

## Data Availability

The data that support the findings of this study are available from the corresponding author A.S. upon reasonable request.

## Abbreviations

The following abbreviations are used in this manuscript:

PNB	Percutaneous Needle Biopsy
US	Ultrasound
CT	Computer Tomography
MR	Magnetic Resonance
HMD	Head-Mounted Display
PV	Photo–Video
AHAT	Articulated Hand Tracking
AR	Augmented Reality
CBCT	Cone–Beam CT
